# Dietary Fatty Acids Affect Red Blood Cell Membrane Composition and Red Blood Cell ATP Release in Dairy Cows

**DOI:** 10.3390/ijms20112769

**Published:** 2019-06-05

**Authors:** Denis Revskij, Susanne Haubold, Torsten Viergutz, Claudia Kröger-Koch, Armin Tuchscherer, Hermine Kienberger, Michael Rychlik, Arnulf Tröscher, Harald M. Hammon, Hans-Joachim Schuberth, Manfred Mielenz

**Affiliations:** 1 Leibniz Institute for Farm Animal Biology (FBN), Institute of Nutritional Physiology “Oskar Kellner”, Wilhelm-Stahl-Allee 2, 18196 Dummerstorf, Germany; Denis.Revskij@med.uni-rostock.de (D.R.); haubold@fbn-dummerstorf.de (S.H.); viergutz@fbn-dummerstorf.de (T.V.); kroeger-koch@fbn-dummerstorf.de (C.K.-K.); atuchsch@fbn-dummerstorf.de (A.T.); hammon@fbn-dummerstorf.de (H.M.H.); 2 Bavarian Biomolecular Mass Spectrometry Center, Technical University of Munich, Gregor-Mendel-Strasse 4, 85354 Freising, Germany; hermine.kienberger@tum.de; 3 Analytical Food Chemistry, Technical University of Munich, Maximus-von-Imhof-Forum 2, 85354 Freising, Germany; michael.rychlik@tum.de; 4 BASF SE, Chemiestraße 22, 68623 Lampertheim, Germany; arnulf.troesher@basf.com; 5 Immunology Unit, University of Veterinary Medicine, Foundation, Buenteweg 2, 30559 Hannover, Germany; hans-joachim.schuberth@tiho-hannover.de

**Keywords:** dairy cow, red blood cell, dietary fatty acids, n-3 fatty acids, n-6 fatty acids, flotillin-1, pannexin-1, adenosine triphosphate release

## Abstract

Diets of dairy cows are often based on maize silage (MS), delivering lower amounts of n-3 fatty acids (FA) compared to grass silage-based diets. The fatty acid composition of the cell membrane can affect the cell function. We evaluated the effects of an MS-based diet on bovine red blood cell (RBC) membrane FA composition and dietary effects on controlled ATP release of RBC. In trial 1, German Holstein cows were fed an MS-based total mixed ration for 24 weeks. The FA composition of RBC membranes from repeatedly taken blood samples was analysed in addition to the abundance of the RBC membrane protein flotillin-1, which is involved in, for example, cell signalling. In trial 2, four rumen fistulated MS-fed cows were abomasally infused in a 4 × 4 Latin square model with three successively increasing lipid dosages (coconut oil, linseed–safflower oil mix (EFA; rich in n-3 FA), Lutalin®, providing conjugated linoleic acids (CLA) or the combination of the supplements, EFA + CLA) for six weeks, followed by a three-week washout period. In trial 2, we analysed RBC ATP release, flotillin-1, and the membrane protein abundance of pannexin-1, which is involved in ATP release as the last part of a signalling cascade. In trial 1, the total amount of n-3 FA in RBC membranes decreased and the flotillin-1 abundance increased over time. In trial 2, the RBC n-3 FA amount was higher after the six-week infusion period of EFA or EFA + CLA. Furthermore, depending on the dosage of FA, the ATP release from RBC increased. The abundance of flotillin-1 and pannexin-1 was not affected in trial 2. It is concluded that changes of the membrane FA composition influence the RBC function, leading to altered ATP release from intact bovine RBC.

## 1. Introduction

The incorporation of fatty acids (FA) of different chain length and saturation plays a critical role in plasma membrane structure and function [1], and therefore membrane protein activity [2,3,4]. The essential n-3 polyunsaturated FA (PUFA) α-linolenic acid (ALA; 18:3) and the n-6 PUFA linoleic acid (LA; 18:2) cannot be synthesised by mammals. A high plasma n-6:n-3 FA ratio or omega-3 index has been associated with the occurrence of metabolic diseases, inflammation, and other disorders in humans [5,6]. 

Red blood cells (RBC) are often used to investigate membrane lipid composition [7] and membrane protein function [8]. Compared to other tissues, such as adipose tissue, RBCs membranes are easily available and reflect long-term FA intake [9]. RBCs play a major role in meeting the oxygen requirements of tissues. Furthermore, RBCs regulate vascular calibre and therefore blood flow by releasing adenosine triphosphate (ATP) in response to physiological stimuli, such as local oxygen tension and mechanical deformation [10,11]. The signalling mechanisms leading to ATP release include a whole transduction cascade with pannexin 1 (PANX1), a channel forming membrane protein, as the final conduit [12,13]. Metabolic diseases like type 2 diabetes can affect the stimulated ATP release from RBC [14].

Flotillins are ubiquitously expressed integral membrane proteins, including the cell membrane of RBCs. They are involved in several physiological processes, such as endocytosis, trafficking, and signalling [15]. They have a strong potential for self-oligomerization and interaction with other co-localised proteins [16], and they can be considered markers of specific microdomains of the plasma membrane, a structural organization called lipid rafts [15,17]. Recently, compared to healthy controls, a more than four-fold increase of the abundance of flotillin-1 (FLOT1) was observed in RBC membranes of humans with type 2 diabetes [18].

Dietary FA can have modulating effects on plasma membrane and information on the importance of membrane FA composition on membrane associated protein functions, like Ca^2+^-ATPase activity [2], in bovine cells is scarce. Maize silage (MS) is an important component in diets of dairy cows [19,20]. Compared to other dietary components, such as grass/maize silage (GMS), MS delivers high amounts of the n-6 FA LA but lower amounts of the n-3 FA ALA [21,22,23], which possibly modifies physiological functions. For example, the incorporation of n-3 FA in bovine corpora lutea membranes is linked with the formation of membrane lipid microdomains. Higher amounts of n-3 FA lead to the disruption of these microdomains, associated with increased prostaglandin F2α receptor mobility [24]. 

Currently, the minimal n-3 EFA requirement is unknown in dairy cows [25]. A number of studies have been performed to investigate the effects of n-3 or n-6 FA or conjugated linoleic acid (CLA) on the health and efficiency of dairy cows [26,27,28]. However, the effects of FA on RBC function in lactating dairy cows are, at least to our knowledge, unknown. Thus, we hypothesised that the n-3 and n-6 FA composition of RBC membranes in dairy cows responds to changes of the FA pattern in dietary fat and is related to the abundance of plasma membrane-associated proteins linked with ATP release from RBCs. Results of our study may help to characterise potential n-3 essential fatty acid deficiency in dairy cows. Therefore, the objectives of this study were to evaluate the effects of a diet low in n-3 but high in n-6 PUFA content, followed by substitution of a mix of n-6 FA with a high content of n-3 FA and CLA. In our analyses, we focussed on the RBC membrane FA composition and RBC variables linked with cell signalling and oxygen partitioning. We have studied cows in established lactation to avoid potential confounding effects on lipid metabolism due to high levels of endogenous FA derived from lipolysis, as is the case in early lactation.

## 2. Results

### 2.1. Animal Performance Data

*Trial 1*: The body weight of the cows increased over the experimental period, from 601 ± 17 kg at week -1 to 670 kg ± 17 kg (mean ± SE) at week 24 (*p* < 0.01). The dry matter (DM) intake increased from week -2 (16.15 ± 1.01 kg DM/d) to week 24 (20.04 ± 1.10 kg DM/d) (*p* < 0.01). The mean fat intake during the GMS diet feeding was 572.13 ± 1.01 g/d, followed by 440.49 ± 1.02 g/d after the first week of feeding with the MS diet and 488.87 ± 1.10 g/d at week 24. In total, there was a trend for an increase in energy intake (*p* < 0.1) from week -2 (110.04 ± 8.97 MJ/d net energy content for lactation (NEL)) up to week 24 (138.11 ± 9.66 MJ/d NEL). The energy corrected milk (ECM) yield decreased during this time period, from 34.90 ± 2.29 kg/d to 25.65 ± 2.34 kg/d.

*Trial 2*: The mean body weight of the cows was 578 ± 13.9 kg (mean ± SE) at the beginning of the experiment. Although the amounts of supplemented fatty acids differed among supplemental groups in trial 2 (data shown in Supplemental Table S3), there were no differences in DM intake (CTRL: 19.15 ± 1.19; CLA: 17.99 ± 1.19; EFA: 18.96 ± 1.19; CLA_EFA: 18.44 ± 1.19 kg/d, respectively), and energy intake (CTRL: 131.58 ± 8.14; CLA: 123.90 ± 8.17; EFA: 130.32 ± 8.14; CLA_EFA: 126 ± 8.14 MJ NEL/d, respectively) between the supplemental groups over the experimental period. However, the ECM yield differed (CTRL: 21.04 ±2.51; CLA: 16.39 ± 2.52; EFA: 19.54 ± 2.52; CLA_EFA: 18.96 ± 2.51 MJ NEL/d, respectively). The ECM yield in the CLA group was lowest (*p* < 0.05 or lower) compared to all other groups, and tended to be lower (*p* < 0.1) in CLA_EFA than in CTRL.

### 2.2. Effects of an n-3 Fatty Acid Reduced Diet and an Additional Fatty Acid Supplementation on the Fatty Acid Profile of the Plasma Membrane of RBCs

**Figure 1:**
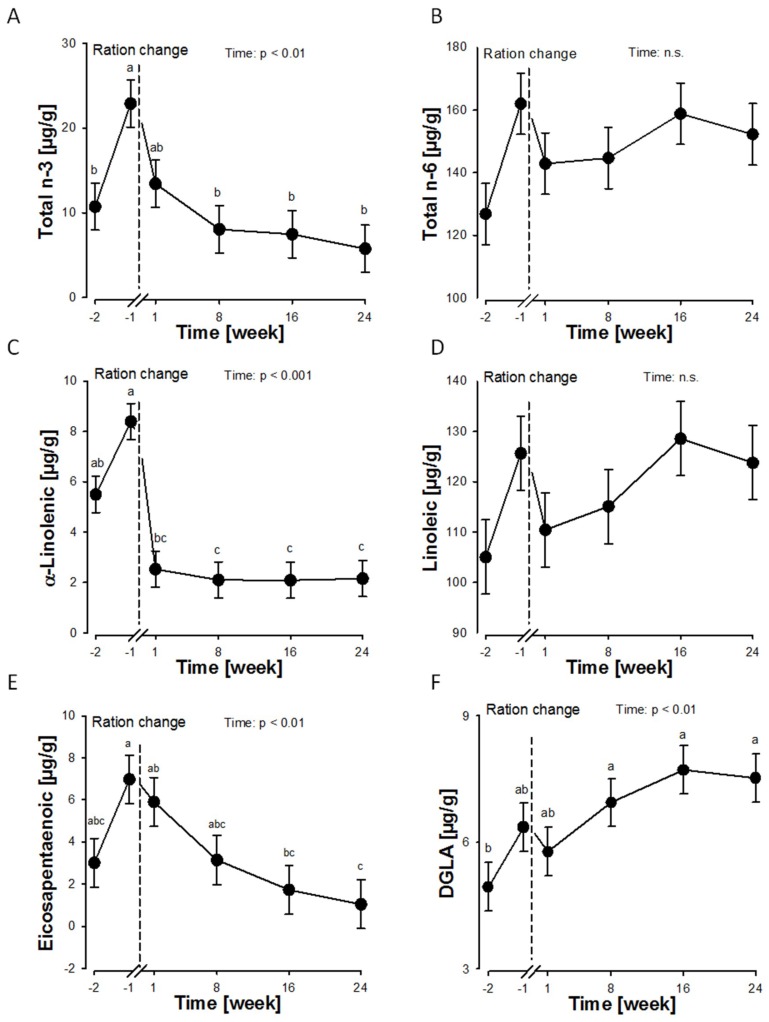
Selected FA (**A**: total n-3 FA; **B**: total n-6 FA; **C**: α-linolenic acid; **D**: linoleic acid; **E**: eicosapentaenoic acid; **F**: dihomo-gamma-linolenic acid (DGLA)) mass fractions (µg/g) of bovine red blood cells plasma membranes over time. The vertical dotted line indicates the change from a mixed grass silage/maize silage-based diet to a maize silage-based ration. Data are expressed as LSM ± SE (*n* = 5). LSM with different letters differ between time points (*p* < 0.05).

In order to analyse changes in the plasma membrane composition of RBCs, caused by a diet low in n-3 FA content (trial 1) and the effects of supplementing FA (trial 2), we isolated RBC plasma membranes from lactating German Holstein cows and determined the total lipid content as well as the RBC fatty acid composition (Figure 1, Table 1, Supplemental Tables S5 and S6).

*Trial 1*: We observed a total n-6:n-3 FA ratio in the bovine RBC membranes of about 8.6:1 for the GMS-based total mixed ration (TMR). The n-6 FA rich maize based TMR (MS) with a total n-6:n-3 FA ratio of 11.4:1 shifted the n-6:n-3 FA ratio of the RBC membranes to more than 26.2:1. After an initial increase from week -2 to week -1, the RBC membrane n-3 FA concentration decreased from week -1 to week 24 (*p* < 0.01), as well as the highest measured n-3 FA α-linolenic acid (*p* < 0.001) (Figure 1A,C). The α-linolenic acid metabolites eicosapentaenoic acid (Figure 1E) and docosapentaenoic acid also decreased from week -1 to week 24 (*p* < 0.01). The amount of total n-6 FA, as well as the n-6 FA linoleic acid did not change during this time period (Figure 1B,D), but the amount of the n-6 FA dihomo-gamma-linolenic acid (DGLA) increased over time (*p* < 0.01) (Figure 1F). The *cis-9, trans-11* CLA concentration decreased between week -2 and week 1 (4.9 µg/g versus 2.3 µg/g; *p* < 0.05), as well as the *trans-10, cis-12* CLA between week -1 and week 1 (29 µg/g *p* < 0.05). The amount of oleic acid (*p* < 0.001) and myristic acid (*p* < 0.05) decreased over time, and the total trans-FA (18:1 *trans-9* + 18:1 *trans-10 + trans-11*) first increased between week -2 and week 1 (*p* < 0.05) and then decreased up to week 24 (*p* < 0.01).

*Trial 2*: At the end of the supplementation period (week 6) the RBC n-3 FA mass fractions were higher in the EFA and EFA + CLA groups compared to the samples which were taken after the three weeks washout (WO) phase (*p* < 0.01) (Table 1). Additionally, the EFA group showed higher n-3 FA levels after an infusion of dosage III compared to the control supplement (*p* < 0.05). The quantity of ALA after the infusion of dosage III was higher in the EFA containing groups than in the control group (*p* < 0.001) and CLA group (*p* < 0.01). In addition, after supplementation of EFA or EFA + CLA, more ALA was incorporated in the RBC membranes compared to the WO phase (*p* < 0.001). However, the control supplement showed more ALA within the WO phase compared to dosage III (*p* < 0.05). The amount of the n-3 FA docosapentaenoic acid was higher in the EFA + CLA group in relation to the WO phase (*p* < 0.05). The quantity of DGLA was lower in the EFA group compared to the WO phase (*p* < 0.05). Furthermore, there was a trend for a treatment x dosage effect for DGLA (*p* = 0.082). In addition, more *trans-10, cis-12* CLA was observed within the EFA + CLA group compared to the WO phase, which was also true for nervonic acid (*p* < 0.05).

### 2.3. Flotillin-1 and Pannexin-1 Abundance in RBC Membranes

In both trials, plasma membranes were extracted from isolated RBCs and the abundance of the membrane protein FLOT1 was analysed by Western blotting (data are provided in Supplemental Tables S7 and S8).

**Figure 2:**
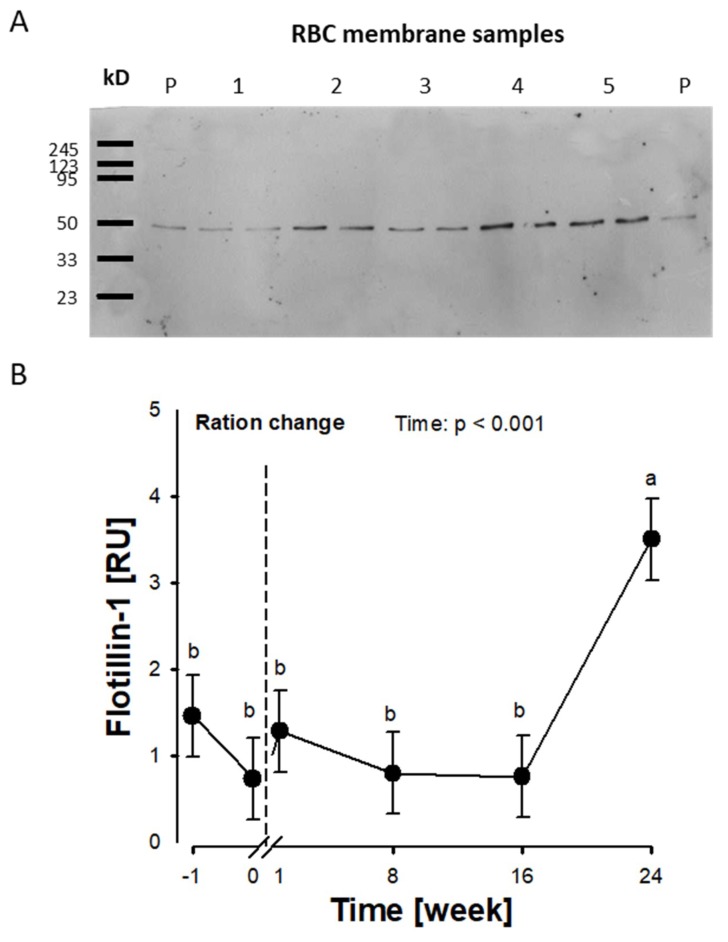
Flotillin-1 (FLOT1) protein abundance in RBC membranes of dairy cows fed a maize-based diet over a time period of 24 weeks (trial 1). FLOT1 protein abundance was analysed using Western blotting. (**A**) Western blot of FLOT1 demonstrating monomers of the protein (individual samples were analysed as duplicates). (**B**) FLOT1 protein abundance over time. Data are expressed as LSM ± SE (*n* = 5); P = pool sample, RU = relative fluorescence units. LSM with different letters differ between time points (*p* < 0.01).

*Trial 1*: Mostly FLOT1 multimers were observed in this trial by our protocol (Supplemental Figure S1). We found an increase of FLOT1 between week 16 and week 24 (*p* < 0.01; Figure 2A,B).

**Figure 3:**
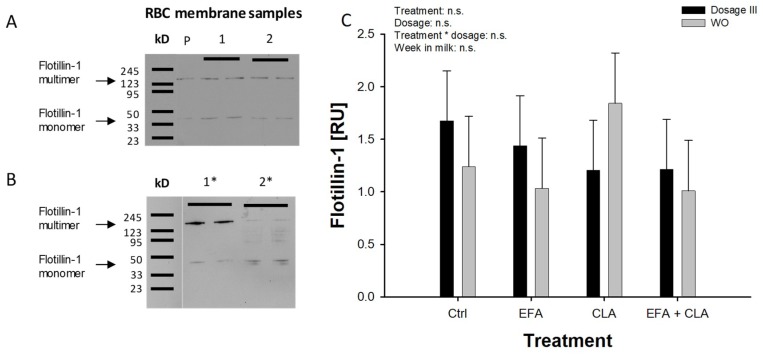
Flotillin-1 (FLOT1) protein abundance in RBC membranes of dairy cows in trial 2. Dairy cows were fed a maize silage-based diet low in n-3 fatty acids and were supplemented with four different FA treatments in three successively increasing dosages using a Latin square design. Data was analysed after supplementing the highest dosage (dosage III) and after the corresponding washout period. (**A**) Representative Western blot of FLOT1; samples were analysed as duplicates. (**B**) FLOT1 multimer formation was confirmed using an extended heat denaturation step (line 1*, 5 min at 95 °C versus line 2*, 20 min at 95 °C). (**C**) Analysed data of trial 2 are presented in figure C. Data are expressed as LSM ± SE (*n* = 4); P = Pool sample; RU = relative fluorescence units.

*Trial 2*: FLOT1 showed a formation of multimers in addition to monomers after SDS-PAGE and Western blotting (Figure 3A), using the same protocol for denaturation, which was confirmed by using an extended heat denaturation step (20 min at 95 °C). The intensity of the multimeric bands was reduced and the intensity of the monomeric FLOT1 bands was increased (Figure 3B). No treatment or time effects were observed in this trial (Figure 3C; additional examples for Western blot of FLOT1 are demonstrated in Supplemental Figures S2 and S3).

Comparable to FLOT1, PANX1 abundance did not show any treatment or time effects in trial 2 and was not measured in trial 1.

### 2.4. ATP Release from Bovine RBC

**Figure 4:**
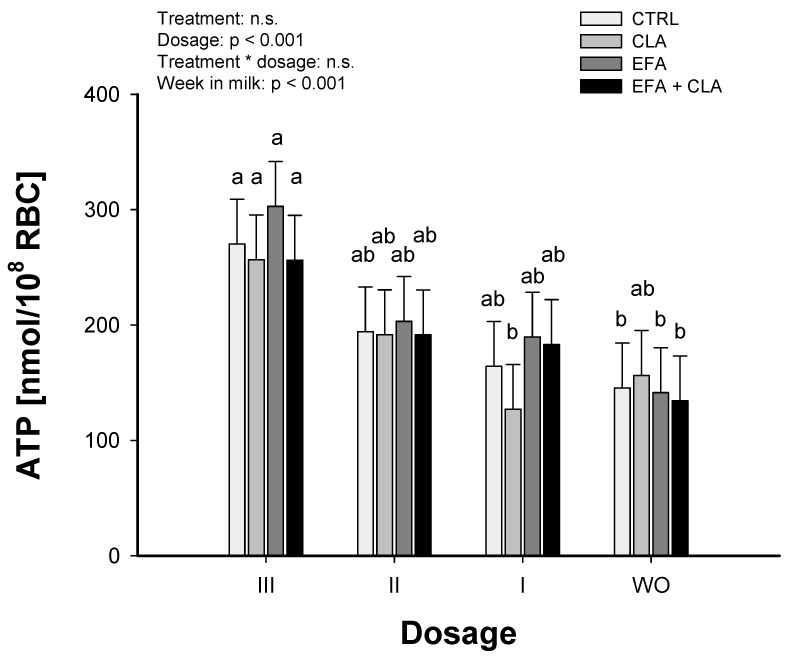
ATP release from bovine red blood cells (RBC), measured in trial 2. Dairy cows were fed a maize silage-based diet low in n-3 fatty acids and were supplemented with four different FA treatments in three successively increasing dosages (I, II, III) using a Latin square design, followed by a three-week washout period (WO). Blood samples were collected and the basal ATP release from isolated RBC was analysed. CRTL = coconut oil consisting of 93% of saturated FA (38.3 g/d in dosage I); EFA = a mix of linseed and safflower oil (39.1 and 1.6 g/d each in dosage I); CLA = Lutalin ®, providing the same amounts of *cis-9*, *trans-11*, and *trans-10*, *cis-12* CLA (16 g/d in dosage I); EFA + CLA = a combination of the EFA and CLA treatment (40.7 g/d + 16 g/d in dosage I). The initial dosage I was doubled every two weeks (dosage II, dosage III), resulting in a six-week supplementation period for each FA supplement. Data are expressed as LSM ± SE (*n* = 4). LSM with different letters differ between dosages within a fatty acid treatment (*p* < 0.05).

*Trial 2*: The basal ATP release was not affected by any FA treatment, but showed a dosage effect (*p* < 0.001). CLA treatment increased (*p* < 0.05) the ATP release in dosage III compared to dosage I. The control, EFA, and EFA + CLA treatments showed a greater ATP release with dosage III compared to the WO period (*p* < 0.05; Figure 4). This difference of ATP release was greatest in the EFA-treated cows (*p* < 0.01). Data on ATP release are provided in Supplemental Table S8.

**Figure 5:**
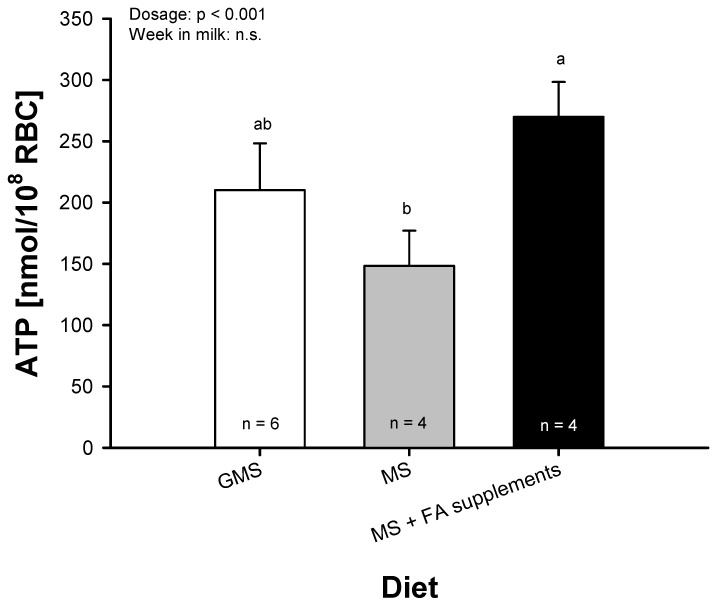
Comparison of the basal ATP release from RBC of dairy cows fed a mixed grass and maize silage-based TMR (GMS) to those fed a maize silage-based TMR (MS). For the analysis of the ATP release of the MS group, data from the end of the washout phase was used and compared with the cows after feeding the highest dosage of fatty acid (FA) supplements (MS + FA supplements). Data of the different FA treatments was merged and are expressed as LSM ± SE. LSM with different letters differ between groups (*p* < 0.05).

All FA treatments at dosage III, with the exception of CLA, led to a greater ATP release compared to the WO period, but no differences were found between the treatment groups. Therefore, after merging the data, the quantified ATP release at the end of the WO period within the MS group and after supplementation of dosage III (MS + FA supplements) of all FA treatments was compared to randomly chosen dairy cows who were fed the GMS diet. This comparison showed a greater ATP release after supplementing the highest dosage (dosage III) compared to the WO (*p* < 0.001), but no differences between GMS and MS TMR were found (Figure 5).

The in vitro incubation of RBC with LA or ALA in different concentrations showed no treatment or dosage effects after stimulation with mas7; only a time effect was observed (*p* < 0.001), resulting in a markedly reduced release of ATP after 8 h of incubation. The stimulation of ATP release with mas-7 in the bovine was highly dependent on the osmotic pressure. No effect was observed with an osmotic pressure of 320 mOsm/L compared to 290 mOsm/L.

## 3. Discussion

We hypothesised that a diet low in n-3 FA content affects variables which are associated with the ATP release of RBC. We therefore analysed the consequences of a low n-3 FA intake, and abomasal supplementation with different fatty acids on bovine RBC fatty acid composition, ATP release, as well as proteins associated with RBC function (FLOT-1) or directly with ATP release (PANX-1).

The n-6:n-3 FA ratio of the bovine RBC membrane for the GMS based TMR was similar to data from a study of Dänicke et al. [29], in which a comparable ration with 25% grass silage was fed. We observed a threefold increase of the RBC plasma membrane n-6:n-3 FA ratio due to the maize-based TMR being high in n-6 FA but low in n-3 FA. In pigs, a low dietary n-6:n-3 FA ratio is associated with a lower abundance of inflammatory proteins like tumor necrosis factor-α [30]. Conversely, a diet with a high n-6:n-3 FA ratio has been linked with metabolic and inflammatory diseases in humans and affects metabolism towards obesity [31]. To the best of our knowledge, comparable data is not available for the bovine.

In humans, the biosynthesis of n-6 and n-3 long chain PUFA is mediated by the Δ-6 desaturase, with higher desaturase activity for n-3 than for n-6 FA [32]. Therefore, low availability of n-3 PUFA may increase the synthesis of n-6 FA also in bovines as was observed for DGLA in trial 1 of the present study.

In trial 1, the total n-3 FA increased in RBC membranes between week -2 and week -1. However, this observation cannot currently be conclusively explained.

In trial 2, the two EFA-containing oil supplements led to an increased incorporation of total n-3 FA in RBC membranes, as expected. However, we found higher ALA concentrations after the WO compared to the related treatment period with coconut oil. We therefore suggest that, different to humans [33], more than three weeks of supplementation are necessary to reduce RBC membrane FA levels and/or other endogenous stores of this FA efficiently in bovines. For future studies on this issue in dairy cows, it should be discussed whether the supplementation periods need to be extended. LA and ALA are precursors for CLA [34] and dairy products are an important source of CLA. Health benefits of CLA for humans are discussed [34] and the improvement of the metabolic status of cows by CLA was described [35,36]. Interestingly, in RBC membranes extracted in samples from trial 1, both CLA isomers *trans-10,cis-12* and *cis-9,trans-11* CLA tended to be reduced by the MS diet and *trans-10,cis-12* CLA was enriched by the EFA + CLA diet in trial 2. The increase of only the *trans-10, cis-12* CLA isomer by the EFA + CLA supplement indicates a different incorporation efficiency of the two CLA isomers into bovine RBC membranes, which was observed in other bovine tissues before [37].

Flotillins are integral membrane proteins involved in several physiological processes, like endocytosis, trafficking, and signalling [15]. FLOT1 is involved in glucose transporter translocation towards the plasma membrane [38,39]. In type 2 diabetic humans, an increase of FLOT1 within the RBC membrane was observed [18]. We observed an increase of FLOT1 in trial 1 between week 16 and 24 after the onset of feeding the MS diet. This observation could be linked with changes of the RBC function in cows receiving the diet low in n-3 FA over time and should be analysed in future.

In trial 2 we did not find any effect of FA supplements on FLOT1, which may depend on the duration of the experimental periods used in both trials and the lifetime of bovine RBCs, which is about 130 to 160 days [40]. In trail 1 and trial 2 mainly tetrameric FLOT1 was detected after Western blotting, which was confirmed by an extended denaturing step. The lipid raft marker FLOT1 forms homo-multimers and hetero-multimers with flotillin-2. The multimers are of importance for the stabilization of microdomains [41].

The productive lifetime of dairy cows is rather short compared to the human lifetime. Therefore, potential negative effects due to an imbalanced n-6:n-3 FA ratio might be more relevant in humans compared to cows over time. Thus, our observation of FLOT1 upregulation due to an unusually high n-6:n-3 ratio within the RBC membrane is possibly of higher importance for human nutrition than for the metabolic health of dairy cows.

The ATP-induced increase in blood flow is linked with the regulation of the vascular calibre [12] and requires a controlled and regulated release by RBCs. To detect functional changes in RBCs we analysed the basal ATP release from RBCs in trial 2 ex vivo, in addition to the abundance of PANX1, the postulated final ATP conduit within the RBC membrane in response to low O_2_ tension [11,42].

In the present study, ATP release by the MS-fed cows was increased by the greatest dosage of the infused fatty acids. The detected differences in basal ATP release could be associated with dietary effects on the membrane fluidity. It was observed that the RBC membranes of miniature pigs supplemented with maize oil had a greater fluidity than RBC membranes of pigs supplemented with significant amounts of n-3 FA derived from fish oil, which was explained by an induced array of the membrane components due to multiple double bounds in the n-3 FA [43]. Our observations in trial 2 might be of importance regarding the oxygen and nutrient supply of dairy cows’ mammary glands by regulating the capillary calibre during lactation. The daily milk yield during the first three months of lactation is correlated with the blood flow volume, as shown by Berger et al. [44]. As discussed by these authors, oxygen and nutrient supply could be affected by the diameter of the pudendoepigastric trunks or by resistance to blood flow. On the other hand, our experimental design could be of interest with regard to human nutrition. The importance of a well-balanced dietary ratio of n-6:n-3 FA or the incorporation of individual FA was demonstrated on the level of the RBC plasma membranes. The increase in ATP release from RBCs is discussed by Forsyth et al. [45] as an approach to improve cardiovascular diseases, diabetes, and blood storage, the latter being associated with reduced ATP release. Comparable to FLOT1, the PANX1 abundance did not change with the different FA supplements in trial 2. These findings, together with an increased ATP release caused by any FA treatment, suggest that the increased basal ATP release was not mediated by the incorporation of additional PANX1 protein into the cell membrane, but could be linked with increased PANX1 activity. Furthermore, it could indicate the involvement of other existing ATP conduits [13], which was established recently regarding to the voltage-dependent anion channel [46]. Our observation of increasing constitutive ATP release in response to the diet indicates the involvement of the ATP conduit, which is activated by prostaglandin I2 and comprises the voltage dependant anion channel. This pathway increases the bulk blood flow without selective improvement of blood flow in regions of reduced O_2_ tension, as discussed by Ellsworth et al. [11]. The time period necessary for FA incorporation in RBC membranes in vitro is relatively short and can reach a plateau phase after 4–6 h, as shown by the use of radioactive labelled FA in vitro [47]. However, we were not able to demonstrate changes of stimulated ATP release in our in vitro system, in which we incubated RBCs with LA or ALA for 4–8 h. Handling and media conditions play a critical role in analysing a controlled ATP release from intact RBC. It was discussed that haemolysis is the primary release mechanism for ATP [48]. In accordance with other studies [49,50,51], we used an isotonic medium for the preparation of RBC and checked the osmolarity of the media as well as the concentration of free haemoglobin in our RBC preparations. Based on our results we could not find any indication of haemolysis.

## 4. Materials and Methods

All experimental procedures in the present study were performed according to the German animal care and protection regulations and were approved by the authorities of the state of Mecklenburg-Vorpommern, Germany (Landesamt für Landwirtschaft, Lebensmittelsicherheit und Fischerei Mecklenburg-Vorpommern, Germany; trial 1: LALLF M-V/ TSD/7221.3-1-043/14 from 22 September 2014; trial 2: LALLF M-V/ TSD/7227.3-1-061/14 from 05 January 2015).

### 4.1. Animals and Study Design

Animal housing and feeding, as well as study conditions for trial 1 and trial 2, were described by Weber et al. [23], and Haubold et al. [52], respectively. The individual dry matter (DM) intake of the cows was recorded. In trial 1, lactating German Holstein cows (*n* = 5, second lactation; 10,000 kg milk in 305 d in first lactation; 57 days postpartum ± 4 at the start of the study) were kept in a tied-stall barn at the Leibniz Institute for Animal Biology (FBN; Dummerstorf, Germany). They had access to water and feed for ad libitum intake, according to the German Society of Nutrition Physiology (GfE) [53,54]. The ECM was calculated according to Reist et al. [55]: ECM (kg) = (0.038 × g of crude fat + 0.024 × g of CP + 0.017 × g of lactose) × kg of milk/3.14. The cows were investigated for 24 weeks after changing from a GMS TMR to an MS TMR, as previously described by Weber et al. [23]. Briefly, diets were largely isoenergetic (7.0 versus 6.9 MJ net energy of lactation (NEL)/kg of dry matter (DM)) and isonitrogenous (GMS: 165 g/kg DM; MS: 158 g/kg DM), but crude fat content was lower in MS than in GMS (24.4 versus 33.1 g/kg DM, respectively). The dietary content of LA was similar (11.7 and 10.8 g/kg DM), but the ALA content was much lower in the MS than in the GMS diet (1.0 versus 6.2 g/kg DM). Dietary ingredients used in trial 1 were in accordance with Weber et al. [23] and are shown in Supplemental Table S1.

**Figure 6:**
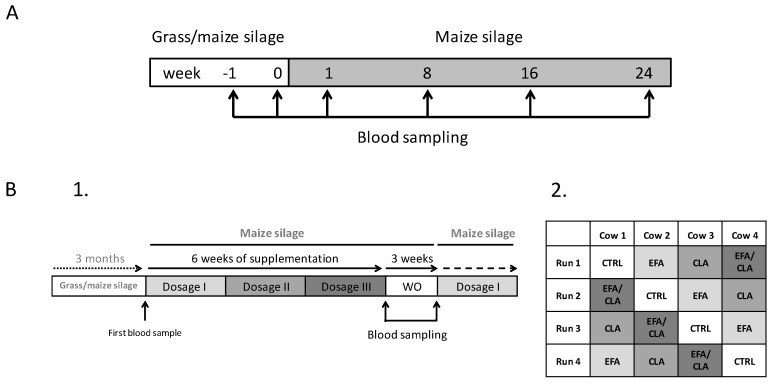
Study design of trial 1 (**A**) and trial 2 (**B**). (**A**) Feeding regime and the time of sampling in relation to the dietary switch (grass/maize versus maize-based silage) over the whole experiment. (**B1**): Demonstration of the study design in relation to the increasing dosages of the FA supplements after switching from a grass/maize to a maize silage-based ration. Each experimental period consisted of a six-week supplementation period followed by a three-week washout period (WO). The initial dosage (dosage I) was doubled every two weeks (dosage II, III). After the WO period, the FA supplement was changed according to Latin square design shown in Figure (**B2**). CRTL = coconut oil consisting of 93% of saturated FA (38.3 g/d in dosage I); EFA = a mix of linseed and safflower oil (39.1 and 1.6 g/d each in dosage I); CLA = Lutalin ®, providing the same amounts of *cis-9, trans-11* and *trans-10, cis-12* CLA (16 g/d in dosage I); EFA + CLA = a combination of the EFA and CLA treatment (40.7 g/d + 16 g/d in dosage I).

In trial 2, German Holstein cows (*n* = 4; third lactation, >10,000 kg milk in 305 d in second lactation; 126 postpartum ± 4 at start of the study) were fitted with an abomasal infusion tube via a rumen fistula and were kept in a free-stall barn at the Leibniz Institute for Animal Biology (FBN; Dummerstorf, Germany). The use of the abomasal infusion tube via the rumen fistula was necessary because of biohydrogenation of fatty acids by ruminal fermentation. The animals had unrestricted access to water and received the MS-based TMR (6.7 MJ NEL/kg of DM; crude protein and fat content: 149, 24 g/kg DM, respectively) with a low n-3 FA content for ad libitum intake, starting three months before the initiation of trial 2 (Supplemental Table S2). The cows were arranged in a 4 × 4 Latin square design and were supplemented with four different FA treatments. The treatment period for each FA supplement consisted of six weeks, during which three different dosages were administered for two weeks each, followed by a three week WO period. The FA supplements were: 1) coconut oil, which mainly (93%) consisted of saturated fatty acids (SFA) (CTRL, 38.3 g/d; Sanct Bernhard, Bad Ditzenbach, Germany); 2) a mix of linseed (DERBY, Derby Spezialfutter GmbH, Münster, Germany) and safflower oil (GEFRO, Memmingen/Allgäu, Germany) (EFA, 39.1 and 1.6 g/d each) delivering 52.3% of n-3 FA and 17.4% n-6 FA (resulting in an n-6:n-3 FA ratio of 1:3); 3) Lutalin®, (CLA, 16 g/d; BASF, Ludwigshafen, Germany; providing 4.6 g/d of cis-9, trans-11 and trans-10, cis-12 CLA); and 4) the combination of EFA and CLA (EFA + CLA; 40.7 g/d + 16 g/d). The initial dosage was doubled every two weeks, resulting in a six-week period for each FA supplement (details on the individual dosages and composition of the oils used for abomasal infusion are shown in Supplemental Tables S3 and S4. The daily amount of FA supplements was split into two equal portions and infused manually via the abomasal infusion tube after milking at 0700 and 1600. Coconut oil was chosen as a control because of its high content of SFA and very low PUFA content (0.9%), in combination with a low melting point at 24 °C, which facilitates the administration via an abomasal tube system. The study design of both trials is shown in Figure 6.

### 4.2. Blood Sampling and Plasma Membrane Extraction of RBC

*RBC Isolation:* Blood samples were drawn from the jugular vein by venipuncture after morning milking, before fatty acid supplementation and feeding. For the analysis of RBC membranes, fat content, as well as FLOT1 and PANX1, lithium heparinate containing tubes (BD Vacutainer, Becton, Dickinson and Company, Plymouth, UK) were used. In trial 1, blood samples were taken in weeks -2 and -1, before the change to the MS diet, and weeks 1, 2, 8, 16, and 24 of the study. In trial 2, blood samples were taken at the end of the six-week supplementation period and at the end of the three-week WO period. Blood samples were placed on ice and processed within 2 h of sampling. Blood samples were centrifuged at 2200× *g* for 15 min at 4 °C. The supernatant was discarded and RBCs were resuspended and washed three times (2200× *g* for 10 min at 4 °C) in Tris-buffered saline (TBS). After a final wash, the TBS supernatant was discarded. This resulted in a concentrated preparation of RBCs with a haematocrit of 95–99%. To prevent FA oxidation, 10 µL of 2% 3,5-di-tert-butyl-4-hydroxytoluene (BHT) was added to 2 mL concentrated RBC and stored at −80 °C until analysis.

*RBC Plasma Membrane Extraction:* RBC membrane extraction was performed as described previously [56], with slight modifications. Concentrated RBCs (2 mL) were thawed on ice and lysed in 40-fold volume (*w/v*) of 5 mM Tris-HCl, 5 mM KCl, pH 7.4 (5T5K buffer) containing 0.1 mM phenylmethylsulfonyl fluoride (PMSF) as a proteinase inhibitor for 10 min on ice, followed by centrifugation at 23,741× *g* for 15 min at 4 °C. The supernatant was discarded and washing steps were repeated two times without the incubation step. Membrane pellets were transferred into a fresh reaction tube. A three-fold volume (w/v) of 5T5K buffer, containing PhosSTOP (Sigma-Aldrich, Schnelldorf, Germany), was added and the pellets were properly homogenized using a pestle (GE Healthcare Bio-Science Corp., New Jersey, USA) and a cannula (26G, B. Braun Melsungen AG, Melsungen, Germany). Membrane samples were stored at −80 °C until analysis.

### 4.3. Fatty Acid Analysis of Total Membrane Lipids

Total lipids were extracted from RBC membranes according to procedures previously described [57], with modifications. Briefly, RBCs were lysed and total RBC membrane lipids were extracted by adding a mixture of chloroform and methanol (2:1, *v/v*), containing 0.01% BHT and processed (1 min, 40 Hz at RT) using an ultrasound sonotrode (type UW 2070, Bandelin, Berlin, Germany). Thereafter, a second volume of a 1:1 (*v/v*) chloroform and methanol mixture was added and processed in the same manner. After centrifugation (4000× g for 5 min at 4 °C), the supernatant was collected and the residue was processed again and both supernatants were combined. Finally, aqueous sodium chloride solution (0.1 mol/L) was added and the solution was mixed for 1 min. After separation, the chloroform layer was removed and evaporated at 37 °C under vacuum. Total lipid extracts were dissolved in methyl-tert-butyl ether and methylated with trimethylsulfonium hydroxide. Fatty acid methyl esters (FAME) were analysed on a Hewlett Packard 6890 gas chromatograph equipped with an HP 6890 Series Mass Selective Detector (Agilent Technologies, Boeblingen, Germany). Peaks were identified by comparing retention times and mass spectra of FAME reference compounds [57].

### 4.4. RBC Isolation and Measurement of ATP Release

In trial 2, the isolation of RBCs was performed as described by Sprague et al. [14], with some modifications. Blood samples were obtained from the jugular vein by venipuncture using potassium EDTA-containing vacutainers (Vacuette Greiner Bio-One International AG, Frickenhausen, Germany). Samples were placed on ice and processed within 2 h of sampling. From each animal, 10 mL blood samples were centrifuged at 500× *g* for 10 min at 4 °C. The plasma and buffy coat were discarded and RBCs were washed three times in buffer with optimized osmolarity for bovine samples containing 4.23 mmol/L KCl, 1.8 mmol/L CaCl_2_, 126.45 mmol/L NaCl, 1.08 mmol/L MgSO_4_, 18.9 mmol/L Tris-HCl, 5.5 mmol/L glucose, and 0.5% BSA—final pH 7.4 and 290 mOsm/L in a total volume of 15 mL. After the final washing, the supernatant was removed, which resulted in a concentrated preparation of RBCs with a haematocrit of 60–80%. Aliquots of the supernatant were collected for the detection of free haemoglobin, as explained below, and stored on ice until analysis. The RBCs were stepwise diluted with wash buffer to achieve a haematocrit of 2%. From each dilution step, aliquots were collected to detect free haemoglobin as indicator for haemolysis, as is usual in this field of work [42]. To measure the ATP release from the RBCs, the 2% RBC dilutions were further diluted—1:50, 1:100, and 1:200—to achieve a haematocrit of 0.04%, 0.02%, and 0.01%, respectively. Three dilution series were chosen as a systematic control. Data on haematocrit measurements for the 2% RBC dilutions are shown in Supplemental Table S9. The concentrations of the dilutions were verified using a Neubauer improved counting chamber (Brand GmbH + CO.KG, Wertheim, Germany). The mean value of the released amount of ATP per 1 × 10^8^ RBC/mL from all three dilutions was calculated and used for the statistical analyses.

Released ATP from freshly isolated RBC was measured in the buffer within 1 h after preparation using luminescence (ATP Kit SL, BioThema, Handen, Sweden). ATP measurement was performed in white 96 microplates (BRANDplates®, 96-well, pure Grade, Brand GmbH + CO.KG, Wertheim, Germany) following the manufacturer’s instruction. Briefly, 120 µL of Tris-EDTA buffer were mixed with 40 µL of each RBC suspension and added as triplicates on a microplate. To start the reaction, 40 µL of the ATP Reagent SL was added. After measuring the light emission in a Fluostar-Optima microplate reader (BMG-Labtech, Ortenberg, Germany) of the sample (smp), 10 µL of ATP standard (std) was added to each well and light emission (I) was measured again. ATP concentration in each well was calculated by the following equation: ATP_smp_ = 10^−7^ * I_smp_ / (I_smp+std_ − I_smp_). The factor 10^−7^ indicates the concentration of the ATP standard in mol/L. The amount of released ATP was corrected for dilution and normalized to a cell count of 1 × 10^8^ RBC/mL.

To ensure samples were not haemolysed, free haemoglobin was determined after centrifugation at 500 × *g* for 10 min and 4 °C by light absorption (Sunrise^TM^, TECAN, Switzerland) at a wave length of 415 nm. Blood smears of washed RBCs were analysed microscopically for platelet contamination using Pappenheim staining.

### 4.5. Stimulated ATP Release from RBC in vitro, Incubated with LA and ALA

Blood samples were collected from four randomly selected dry dairy cows not identical with the experimental cows after sacrification, placed on ice and processed within 2 h of sampling. From each animal, three 50 mL blood samples were centrifuged at 500× *g* for 15 min at 4 °C. The plasma and buffy coat were discarded and the RBCs were washed two times in buffer, as described above. A third washing step was performed using a buffer containing 1% fatty acid free BSA (Alfa Aesar, Thermo Fisher Scientific, Karlsruhe, Germany). After the final washing, the supernatant was removed, resulting in an RBC preparation with a haematocrit of 60–80%, which was further diluted to a haematocrit of 20%. Aliquots of 2 mL of this dilution were transferred into a six-well culture plate. The RBCs were further diluted 1:2 with a buffer containing 1% fatty acid free BSA and α-linolenic acid or linoleic acid (50 µM, 200 µM, 400 µM). Controls were incubated with the buffer but without fatty acids (NTC = no treatment control) and with the buffer containing only the solvent ethanol (SC = solvent control). After preparation of the RBC suspension in adequate concentrations, cells were placed on a rocker (MINI-Rocker, Kisker Biotech GmbH & Co. KG, Steinfurt, Germany; angle = 7°, frequency = 30 rpm) and incubated for 4 h and 8 h at 37 °C in a humidified atmosphere with 5% CO_2_. After incubation, the RBC solution was collected and washed (500 × *g* for 10 min at 4°C) two times with a buffer containing 1% fatty acid free BSA. The RBCs were stepwise diluted with the buffer to achieve a haematocrit of 0.04%. The release of ATP was stimulated by the incubation of RBCs with 50 µM mas7 (antibodies-online, Aachen, Germany) for 30 min at 37 °C in a humidified atmosphere with 5% CO_2_. Immediately after incubation, the samples were placed on ice and the released ATP was measured as above.

### 4.6. SDS-PAGE and Western Blot Analysis

For immunoblot detection of the RBC membrane associated proteins FLOT1 in samples of trial 1 and 2 and PANX1 in samples of trial 2, extracted membranes were thawed on ice and protein concentration was determined using the bicinchoninic acid (BCA) assay (Thermo Fisher Scientific, Schwerte, Germany). For protein detection, 10 µg (FLOT1) or 20 µg (PANX1) of the total membrane protein were used. The protein was solubilized and denatured in a loading buffer with 2% SDS (95 °C, 5 min) and transferred to nitrocellulose membranes after SDS-PAGE. Membranes were exposed to the polyclonal rabbit anti-FLOT1 and anti-PANX1 antibody (both Aviva Systems Biology, San Diego, CA, USA; order no.: ARP65758_P050, ARP42783_P050, respectively) overnight at 4 °C, in a dilution of 1:500 in a TBST buffer containing 3% BSA (FLOT1) or a TBST buffer with Triton X-100 instead of Tween-20 (PANX1). After rinsing, the membrane was incubated with a horseradish peroxidase (HRP) labelled secondary anti-rabbit IgG antibody (FLOT1, 1:10,000; Santa Cruz Biotechnology, Heidelberg, Germany) or WestVision™ anti-rabbit IgG antibody for PANX1 (1:10,000, Vector laboratories, Burlingame, CA, USA) for 60 min at RT. Antigen–antibody immunocomplexes were visualized using an enhanced chemiluminescence detection system (ECL; Thermo Fisher Scientific, Schwerte, Germany). The produced chemiluminescence signal was captured on films (FLOT1, Thermo Fisher Scientific, Schwerte, Germany). Chemiluminescence of the PANX1 antigen–antibody complexes were visualized using the Azure c600 chemiluminescence system (Azure Biosystems, Dublin, CA, USA). Band intensities for FLOT1 were analysed using ImageQuant TL software (GE Healthcare, Freiburg, Germany; 1D, v.8.1) and for PANX1 using the AzureSpot software (Azure Biosystems, Dublin, CA, USA; v.14.2). To normalize the target protein signal, the membrane was irreversibly stained with Indian ink after chemiluminescence detection. Specific band intensities were normalized against protein loading, according to Ni et al. [58]. This method helps to circumvent problems related to the regulation of potential reference proteins, as shown in dairy cow adipose tissue over time [59]. The intensities of four identical protein bands of each lane were quantified and the mean was used for normalization. The normalized mean intensity of the duplicate bands of the sample in relation to the normalized mean intensity of the pool sample was calculated and used for the statistical analysis.

### 4.7. Statistical Analysis

In trial 1, data of FA mass fractions and membrane protein abundance were analysed by repeated measure ANOVA using the MIXED procedure of SAS 9.4 (SAS Institute Inc., Chicago, IL, USA). The model contained the fixed (repeated) effect time.

In trial 2, FA composition and protein abundance data at the end of the dosage III period and of the last WO week was analysed. In the model, the fixed factors treatment (CTRL, CLA, EFA, CLA+EFA), dosage (WO, I, II, III) and the interaction treatment × dosage were used. Weeks in milk served as covariate. For statistical analysis of ATP release, we analysed data of the second week of each dosage (weeks 2, 4, 6) and at the end of the last WO week. The same statistical model was used to calculate the ATP release after the combination of all treatments, excluding the factor treatment. ATP release of cultured RBCs was analysed using the MIXED procedure of SAS, using a model with the fixed factors incubation time, fatty acid concentration, and mas7 stimulation. For statistical analysis of FA composition and protein abundance, data determined at the highest dosage period (week 6) and of the last WO week were used. Results are presented as least square means (LSM) ± standard error (SE). Differences of LSM were tested using the Tukey–Kramer test. Effects were considered significant at *p* < 0.05.

## 5. Conclusions

An MS-based diet in dairy cows changes the plasma membrane FA composition of RBCs towards a higher n-6:n-3 FA ratio. The increase of the n-6 FA content of the RBC plasma membrane could increase membrane fluidity, leading to reduced constitutive ATP release ex vivo. Further research is required to analyse dietary effects on constitutive but also regulated ATP release, with the aim of understanding the effects of dysregulated ATP release on the oxygen and nutrient supply of highly metabolic active tissues in dairy cows.

## Figures and Tables

**Table 1:** Selected fatty acids (µg/g sample) in RBC membranes of cows fed a maize based TMR and supplemented with different fatty acids for six weeks, analysed after supplementation of the highest dosage (dosage III) and a three week washout period in trial 2.

		Treatment					*P*-Value			
Fatty Acid in RBC Membranes (µg/g)	ω	CTRL ^1^	CLA ^2^	EFA ^3^	CLA + EFA ^4^	SE ^5^	Treatment	Dosage	Treatment × Dosage	Time ^6^
18:2, 9-*cis,trans-11*							0.042	0.289	0.248	0.000
Dosage III		4.90	7.01	6.51	5.63	0.85				
Washout		5.81	6.04	6.23	3.83	0.86				
18:2, *trans-10,cis-12*							0.250	0.157	0.290	0.000
Dosage III		0.85	1.59	0.98	1.47 *	0.27				
Washout		0.92	1.24	0.99	0.57	0.27				
*18:3 cis-9, cis-12, cis-15*	n3						0.161	0.008	0.000	0.000
Dosage III		8.32 ^b^	9.69 ^b^	17.25 *^a^	16.56 *^a^	1.34				
Washout		12.51 *	11.47	8.63	8.05	1.35				
20:3 *cis*-8,*cis*-11,*cis*-14	n6						0.401	0.292	0.082	0.192
Dosage III		13.04	11.26	9.41	8.59	1.61				
Washout		10.98	10.46	14.03 *	11.13	1.63				
22:5 *cis*-7,*cis*-10,*cis*-13,*cis*-16,*cis*-19	n3						0.503	0.227	0.108	0.575
Dosage III		6.55	9.81	12.47	12.40 *	3.61				
Washout		8.41	12.61	6.76	3.85	3.63				
24:1, 15c							0.453	0.420	0.224	0.000
Dosage III		0.25	0.49	0.21	0.55 *	0.18				
Washout		0.23	0.50	0.36	−0.01	0.18				
Sum CLA							0.082	0.220	0.228	0.000
Dosage III		5.75	8.60	7.49	7.10 *	1.05				
Washout		6.73	7.28	7.21	4.40	1.06				
Sum n-3							0.583	0.058	0.004	0.108
Dosage III		21.31 ^b^	27.18 ^ab^	37.42 *^a^	35.90 *^a^	4.66				
Washout		28.76	31.04	21.96	18.19	4.69				
Sum n-6							0.696	0.937	0.777	0.751
Dosage III		280.37	289.61	264.85	247.21	33.95				
Washout		268.46	258.45	304.72	242.09	34.30				

^1^ CRTL = coconut oil (Sanct Bernhard, Bad Ditzenbach, Germany; 153.1 g/d in dosage III), mainly (93%) consist of saturated fatty acids, ^2^ CLA = Lutalin ® (BASF, Ludwigshafen, Germany; 64.1 g/d in dosage III; providing the same amounts of *cis-9, trans-11* and *trans-10, cis-12* CLA: 18.4 g/d of each in dosage III), ^3^ EFA = a mix of linseed (DERBY, Derby Spezialfutter GmbH, Münster, Germany; 156.4 g/d in dosage III) and safflower oil (GEFRO, Memmingen/Allgäu, Germany; 6.4 g/d in dosage III), delivering high amounts of n-3 FA, but also some n-6 FA, ^4^ CLA + EFA = a combination of the EFA and CLA treatment, ^5^ SE = standard error, ^6^ Time = statistical covariate weeks in milk, ^7^ Dosage III = highest supplemented treatment dosage, ^8^ Washout = time period without fatty acid treatment. Data are expressed as LSM (*n* = 4). LSM with different letters differ between supplementation groups (*p* < 0.05); * indicate differences between dosage III and washout periods (*p* < 0.05).

## Supplementary Materials

Supplementary materials can be found at https://www.mdpi.com/1422-0067/20/11/2769/s1.
