# Supplementary material for: Dietary Fatty Acids Affect Red Blood Cell Membrane Composition and Red Blood Cell ATP Release in Dairy Cows
Source: Int J Mol Sci. 2019 Jun 5;20(11):2769. doi: 10.3390/ijms20112769 (PMC6600345; doi:10.3390/ijms20112769)
Supplement: Supplementary file 1 [file ijms-20-02769-s001.pdf]

Supplementary Materials

Supplemental Table S1. Ingredients and chemical composition of the control diet (GMS) and the n-3 FA reduced diet (MS) used in trial 1

**Item (g/kg of DM)**	**TMR**	
Ingredients	GMS[5]	MS[6]
Maize silage	351	436
Grass silage	226	
Straw	20	114
Hay	20	
Concentrate MF 2000	207	
Grain of corn[1]	73	
Extracted rapeseed meal[1]	52	
Grain of wheat	20	
Extracted soy bean meal	21	117
Dried sugar beet pulp		214
Grain of rye[1]		105
Minerals[2]		10
Chalk[3]		2
Salt[3]		2
Urea[4]		7
Chemical composition		
NEL, MJ/kg DM	7	6.9
Crude fat	33.1	24.4
Crude fiber	165	171
Crude protein	165	158
Utilizable protein	160	163
Starch	271	239
[5]ADF	198	198
[6]NDF	370	341
18:2 *cis*-9, *cis*-12	11.7	10.8
18:3 *cis*-9,*cis*-12,*cis*-15	6.2	1.0

[1]FUGEMA/ Ceravis AG, Malchin, Germany
[2]Rinderstolz 9522 (Salvana GmbH, Sparrieshoop, Germany) composed the following: 92% crude ash, 20% calcium, 5% phosphorus, 6% magnesium, 8% sodium, and vitamins A, D3, E, copper sulfate, zinc oxide, manganese oxide, calcium iodate, sodium selenite
[3]$CaCO_3$ and NaCl, respectively, (Spezialfutter Neuruppin GmbH & Co.KG, Neuruppin, Germany)
[4]Piarumin, SKW Stickstoffwerke Piesteritz GmbH, Lutherstadt Wittenberg, Germany
[5]ADF = acid detergent fiber
[6]NDF = neutral detergent fiber
GMS = grass silage based total mixed ration
MS = maize silage based ration

Supplemental Table S2. Ingredients, chemical composition and fatty acid composition of the n-3 FA reduced diet used in trial 2

**Item (g/kg of DM)**	
Ingredients	
Maize silage	433
Straw	115
Extracted soy bean meal[1]	124
Dried sugar beet pulp[1]	207
Grain of rye[1]	101
Minerals[2]	10
Chalk[3]	2
Salt[3]	2
Urea[4]	7
Chemical composition	
Crude fat	24
Crude fiber	179
Crude protein	149
Utilizable protein	147
Starch	225
ADF	204
NDF	361
NEL (MJ/kg DM)[5]	6.7
Fatty acid	
12:0	0.02
14:0	0.14
15:0	0.04
16:0	4.89
16:1, *cis*-9	0.05
17:0	0.06
18:0	0.71
18:1, *cis*-9	4.54
18:1, *cis*-11	0.22
18:2, *cis*-9,*cis*-12	9.89
18:3, *cis*-9,*cis*-12,*cis*-15	0.88
20:0	0.16
20:1, *cis*-11	0.10
21:0	0.05
22:0	0.26
22:2, *cis*-13,*cis*-16	0.06
24:0	0.32
Ratio n-6:n-3	11.34

[1]FUGEMA/ Ceravis AG, Malchin, Germany
[2]Rinderstolz 9522 (Salvana GmbH, Sparrieshoop, Germany) composed the following: 92% crude ash, 20% calcium, 5% phosphorus, 6% magnesium, 8% sodium, and vitamins A, D3, E, copper sulfate, zinc oxide, manganese oxide, calcium iodate, sodium selenite
[3]$CaCO_3$ and NaCl, respectively, (Spezialfutter Neuruppin GmbH & Co.KG, Neuruppin, Germany)
[4]CH_4N_2O, (Bergophor Futtermittelfabrik Dr. Berger GmbH & Co.KG, Kulmbach, Germany)
[5]German Society of Nutrition Physiology, 2008

Supplemental Table S3. Composition and amounts of daily abomasally infused supplements[1] in trial 2

	Treatments						
	**CTRL**[2,3]	**EFA**[2]		**CLA**[3]	**EFA+CLA**		
	Coconut oil[4]	Linseed oil[5]	Safflower oil[6]	Lutalin[7]	Linseed oil[5]	Safflower oil[6]	Lutalin[7]
Daily infused amounts (g/d)[8]							
Dosage I	38.3	39.1	1.6	16.0	39.1	1.6	16.0
Dosage II	76.6	78.2	3.2	32.0	78.2	3.2	32.0
Dosage III	153.1	156.4	6.4	64.1	156.4	6.4	64.1
Amounts included in dosage I (g/d)							
18:3 *cis*-9, *cis*-12, *cis*-15	0.0	21.1	0.0	0.0	21.1	0.0	0.0
18:2 *cis*-9, *cis*-12	0.0	5.8	1.2	0.4	5.8	1.2	0.4
18:2 *cis*-9, *trans*-11	0.0	0.0	0.0	4.6	0.0	0.0	4.6
18:2 *trans*-10, *cis*-12	0.0	0.0	0.0	4.6	0.0	0.0	4.6

[1]Cows were investigated in a 4x4 Latin square design and were daily supplemented either with coconut oil (CTRL), linseed and safflower oil (EFA), Lutalin® (CLA, *c9, t11* and *t10, c12*), and EFA+CLA.
[2]Composition of CTRL and EFA supplements were calculated to be isoenergetic.
[3]Addition of Vitamin E (0.03, 0.06, 0.12 g/d for dosage I, II, and III, respectively), Covitol 1360 (BASF, Ludwigshafen, Germany)
[4]Sanct Bernhard, Bad Ditzenbach, Germany
[5]DERBY, Derby Spezialfutter GmbH, Münster, Germany
[6]GEFRO, Memmingen/Allgäu, Germany
[7]BASF, Ludwigshafen, Germany
[8]The initial dose was doubled after 2 and 4 wk, resulting in a 6-wk treatment period with 3 doses (I,II,III) and followed by a 3-wk washout period.

Supplemental Table S4. Fatty acid composition of the oils used for abomasal infusion

Fatty acid (% of total fat)	ω	Coconut oil[1]	Linseed oil[2]	Safflower oil[3]	Lutalin[4]
6:0		0.67	0.00	0.00	0.00
8:0		8.49	0.00	0.00	0.00
10:0		6.58	0.00	0.00	0.00
12:0		48.87	0.00	0.00	0.00
14:0		17.63	0.02	0.10	0.00
15:0		0.00	0.00	0.00	0.08
16:0		7.74	5.51	6.42	5.50
16:1 *cis*- 9		0.00	0.06	0.08	0.07
17:0		0.00	0.05	0.00	0.07
18:0		2.84	3.30	2.74	4.33
18:1 *cis*-9		4.83	20.34	14.67	26.22
18:1 *cis*-11		0.00	0.55	0.63	0.67
18:2 *cis*-9, *cis*-12	*n-6*	0.89	14.90	72.90	2.61
18:2 *cis*-9, *trans*-11		0.00	0.00	0.00	28.91
18:2 *cis*-9, *trans*-12		0.00	0.00	0.40	0.00
18:2 *trans*-10, *cis*-12		0.00	0.00	0.00	28.90
18:3 *cis*-9, *cis*-12, *cis*-15	*n-3*	0.00	53.85	0.10	0.00
20:0		0.06	0.12	0.33	0.25
20:1 *cis*-11		0.00	0.12	0.17	0.54
22:0		0.00	0.11	0.21	0.68
23:0		0.34	0.00	0.00	0.00
24:0		0.07	0.07	0.11	0.17
24:1 *cis*-15		0.00	0.00	0.13	0.00
SFA[5]		93.28	9.18	9.92	11.08
MUFA[6]		4.83	21.07	15.63	27.50
PUFA[7]		0.89	68.75	73.00	2.61
Total CLA[8]		<0.05	0.00	0.00	57.80
trans-fatty acids		<0.05	0.00	<0.05	<0.05
Sum of n-3 fatty acids		<0.05	53.85	0.10	<0.05
Sum of n-6 fatty acids		0.89	14.90	72.90	2.61
Vitamin E		0.00	0.070	0.035	0.00

[1]Sanct Bernhard, Bad Ditzenbach, Germany
[2]DERBY, Derby Spezialfutter GmbH, Münster, Germany
[3]GEFRO, Memmingen/Allgäu, Germany
[4]BASF, Ludwigshafen, Germany
[5]Sum of saturated fatty acids, consisting of 6:0; 8:0; 10:0; 12:0; 14:0; 16:0; 17:0; 18:0; 20:0; 22:0; 23:0 and 24:0
[6]Sum of monounsaturated fatty acids, consisting of 16:1 *cis*-9; 18:1 *cis*-9; 18:1 *cis*-11; 20:1 *cis*-11 and 24:1 *cis*-15
[7]Sum of polyunsaturated fatty acids, consisting of 18:2 *cis*-9, *cis*-12 and 18:3 *cis*-9, *cis*-12, *cis*-15
[8]Sum of total conjugated linoleic acids, consisting of 18:2 *cis*-9, *trans*-11and 18:2 *trans*-10, *cis*-12

Supplemental Table S5. Absolute fatty acid concentrations in the RBC membrane of cows fed a maize based TMR over a period of 24 weeks in trial 1

		Time (week)							*P*-value
**Fatty acid in RBC membranes (µg/g)**	**ω**	**-2**	**-1**	**1**	**8**	**16**	**24**	**SE**[1]	**Time**
14:0		1.46^{ab}	1.57^{a}	1.21^{ab}	1.21^{ab}	1.20^{ab}	1.01^{b}	0.12	0.013
16:0		52.63	56.34	55.72	51.34	56.45	55.53	1.77	0.079
16:1 cis-9		8.27	9.25	9.11	8.69	9.74	9.57	0.91	0.093
18:0		106.51	115.38	112.34	102.16	112.97	111.93	4.12	0.091
18:1 (*trans*-9+*trans*-10+*trans*-11)		1.02^{b}	1.07^{ab}	1.92^{a}	1.15^{ab}	0.83^{b}	0.71^{b}	0.22	0.006
18:1 *cis*-9		277.05^{abc}	302.83^{a}	299.00^{ab}	260.69^{bcd}	253.86^{cd}	236.20^{d}	12.35	0.000
18:1 *cis*-11		4.36	4.81	6.36	4.48	6.27	6.87	0.68	0.060
18:2 *cis*-9,*cis*-12	*n-6*	105.05	125.64	110.45	115.11	128.57	123.76	7.37	0.068
18:2 *cis*-9,*trans*-11		4.99^{a}	5.08^{a}	2.30^{b}	4.08^{ab}	4.33^{ab}	3.56^{ab}	0.55	0.026
18:2 *trans*-10,*cis*-12		2.90^{a}	1.49^{ab}	0.98^{b}	2.14^{ab}	2.84^{ab}	2.73^{ab}	0.46	0.021
18:3 *cis*-6,*cis*-9,*cis*-12	*n-6*	1.50	1.29	0.90	1.66	1.08	0.56	0.28	0.137
18:3 *cis*-9,*cis*-12,*cis*-15	*n-3*	5.50^{ab}	8.40^{a}	2.54^{bc}	2.11^{c}	2.09^{c}	2.16^{c}	0.72	0.000
20:3 *cis*-8,*cis*-11,*cis*-14	*n-6*	4.94^{b}	6.35^{ab}	5.78^{ab}	6.94^{a}	7.72^{a}	7.52^{a}	0.57	0.002
20:4 *cis*-5,*cis*-8,*cis*-11,*cis*-14	*n-6*	15.34	28.70	25.75	20.97	21.43	20.40	4.41	0.151
20:5 *cis*-5,*cis*-8,*cis*-11,*cis*-14,*cis*-17	*n-3*	3.01^{abc}	6.98^{a}	5.90^{ab}	3.14^{abc}	1.73^{bc}	1.04^{c}	1.16	0.002
22:5 *cis*-7,*cis*-10,*cis*-13*cis*-,16,*cis*-19	*n-3*	1.31^{bc}	2.43^{a}	1.79^{ab}	1.48^{abc}	0.95^{bc}	0.67^{c}	0.28	0.001
22:6 cis-4,cis-7,cis-10,cis-13,cis-16,cis-19	*n-3*	0.92	5.08	3.20	1.32	2.70	1.89	1.10	0.128
SFA[2]		160.60	173.29	169.28	154.72	170.62	168.46	4.96	0.076
MUFA[3]		289.67^{abc}	316.88^{a}	314.47^{ab}	273.85^{bc}	269.87^{c}	252.64^{c}	12.75	0.000
PUFA[4]		137.55^{b}	184.88^{a}	156.30^{ab}	152.73^{ab}	166.26^{ab}	158.00^{ab}	11.81	0.094
trans-fatty acids[5]		1.02^{b}	1.07^{ab}	1.92^{a}	1.15^{ab}	0.83^{b}	0.71^{b}	0.22	0.006
Sum n-3 fatty acids		10.72^{b}	22.89^{a}	13.43^{ab}	8.05^{b}	7.47^{b}	5.76^{b}	2.78	0.002

Sum n-6 fatty acids	126.83	161.98	142.87	144.68	158.79	152.24	9.77	0.101

[1]SE = standard error; n = 5

[2]Sum of saturated fatty acids, consisting of 14:0; 16:0 and 18:0

[3]Sum of monounsaturated fatty acids, except of 18:1 *trans*-Isomers

[4]Sum of polyunsaturated fatty acids, except of 18:2, *cis*-9,*trans*-11 and 18:2, *trans*-10,*cis*-12

[5]Sum of *trans*-fatty acids, consisting of 18:1 (*trans*-9+*trans*-10+*trans*-11)

Within fatty acid means labelled with different superscript letters differ over time ($p < 0.05$)

Supplemental Table S6. Absolute amounts of all quantifiable fatty acids in the RBC membrane of cows fed a maize based TMR and supplemented with different fatty acid-treatments for 6 weeks, analysed after a 3 week washout period and after supplementation of the highest dosage (dosage III) in trial 2.

		Treatment					*P*-value			
**Fatty acid (µg/g sample)**	**ω**	**CTRL[1]**	**CLA[2]**	**EFA[3]**	**CLA + EFA[4]**	**SE[5]**	**Treatment**	**Dosage**	**Treatment × Dosage**	**Time[6]**
8:0							0.988	0.711	0.883	0.016
Dosage III[7]		3.20	2.08	2.22	2.07	1.25				
Washout[8]		2.37	3.32	2.59	2.77	1.27				
10:0							0.932	0.541	0.975	0.000
Dosage III		0.60	0.65	0.57	0.66	0.22				
Washout		0.61	0.85	0.72	0.71	0.22				
12:0							0.778	0.600	0.669	0.092
Dosage III		0.71	0.43	0.74	0.93	0.23				
Washout		0.53	0.63	0.73	0.55	0.23				
14:0							0.649	0.537	0.445	0.073
Dosage III		3.68	2.29	3.49	10.78	3.15				
Washout		4.54	3.63	3.33	2.75	3.19				
iso-14:0							0.875	0.746	0.730	0.001
Dosage III		0.30	0.43	0.22	0.30	0.10				
Washout		0.30	0.36	0.49	0.27	0.11				
14:1 *cis*-9							0.656	0.777	0.941	0.000
Dosage III		1.33	0.82	0.93	1.01	0.31				
Washout		1.21	0.99	1.12	1.02	0.31				
anteiso-15:0							0.715	0.599	0.374	0.000
Dosage III		3.12	2.24	4.26	2.44	1.29				
Washout		2.69	3.39	2.52	2.24	1.30				

Table S6 continued

Fatty acid (µg/g sample)	ω	CTRL[1]	CLA[2]	EFA[3]	CLA + EFA[4]	SE[5]	Treatment	Dosage	Treatment × Dosage	Time[6]
		Treatment					*P*-value			
iso-16:0							0.996	0.943	0.636	0.146
Dosage III		0.84	0.89	0.51	1.16	0.48				
Washout		0.97	0.77	1.10	0.45	0.48				
16:0							0.935	0.628	0.538	0.792
Dosage III		55.49	57.00	51.41	97.44	23.63				
Washout		63.65	54.05	63.78	43.45	23.89				
16:1 *cis*-9							0.987	0.842	0.840	0.339
Dosage III		14.82	13.97	15.17	15.99	1.81				
Washout		14.92	15.38	14.15	14.45	1.82				
iso-17:0							0.981	0.536	0.909	0.337
Dosage III		2.68	2.12	2.66	3.18	1.06				
Washout		2.25	2.14	2.48	1.71	1.08				
anteiso-17:0							0.997	0.785	0.887	0.092
Dosage III		4.20	4.53	3.90	4.87	1.70				
Washout		4.29	3.73	4.89	3.12	1.72				
18:0							0.999	0.716	0.786	0.260
Dosage III		117.36	119.98	108.46	145.86	36.34				
Washout		117.48	109.54	131.27	91.66	36.74				
18:1 *trans*-9 (+*trans*-6+*trans*-10)							0.988	0.477	0.869	0.005
Dosage III		6.23	5.72	7.33	7.39	2.30				
Washout		4.92	6.72	5.40	4.46	2.32				

Table S6 continued

Fatty acid (μg/g sample)	ω	Treatment					P-value			
		CTRL[1]	CLA[2]	EFA[3]	CLA + EFA[4]	SE[5]	Treatment	Dosage	Treatment × Dosage	Time[6]
18:1 *trans*-11							0.835	0.521	0.529	0.818
Dosage III		3.23	3.26	2.90	6.87	1.83				
Washout		2.93	3.57	3.61	2.47	1.85				
18:1 *cis*-9							0.980	0.563	0.909	0.159
Dosage III		217.93	226.66	219.38	238.52	23.31				
Washout		223.94	206.20	216.44	213.45	23.56				
18:1 *cis*-11							0.838	0.975	0.898	0.964
Dosage III		7.65	6.70	6.36	7.39	1.29				
Washout		7.66	6.15	7.59	6.83	1.30				
18:2 *cis*-9,*cis*-12	*n-6*						0.823	0.985	0.896	0.798
Dosage III		214.97	217.85	205.61	193.03	31.99				
Washout		209.18	198.68	235.49	186.27	32.34				
18:2 *cis*-9,*trans*-11							0.042	0.289	0.248	0.000
Dosage III		4.90	7.01	6.51	5.63	0.85				
Washout		5.81	6.04	6.23	3.83	0.86				
18:2 *trans*-10,cis-12							0.250	0.157	0.290	0.000
Dosage III		0.85	1.59	0.98	1.47*	0.27				
Washout		0.92	1.24	0.99	0.57	0.27				
18:3 *cis*-6,*cis*-9,*cis*-12	*n-6*						0.943	0.925	0.951	0.550
Dosage III		1.01	0.72	0.82	0.57	0.51				
Washout		0.72	0.70	1.10	0.77	0.52				

Table S6 continued

Fatty acid (μg/g sample)	ω	Treatment CTRL[1]	CLA[2]	EFA[3]	CLA + EFA[4]	SE[5]	P-value Treatment	Dosage	Treatment × Dosage	Time[6]
18:3 *cis*-9,*cis*-12,*cis*-15	*n-3*						0.161	0.008	0.000	0.000
Dosage III		8.32[b]	9.69[b]	17.25[*a]	16.56[*a]	1.34				
Washout		12.51[*]	11.47	8.63	8.05	1.35				
20:0							0.942	0.842	0.611	0.642
Dosage III		1.24	1.05	1.21	1.46	0.33				
Washout		1.17	1.23	1.46	0.88	0.34				
20:1 *cis*-11							0.905	0.765	0.847	0.164
Dosage III		0.99	0.88	0.85	0.98	0.40				
Washout		1.16	0.89	1.31	0.71	0.40				
20:2 *cis*-11,*cis*-14	*n-6*						0.427	0.829	0.293	0.001
Dosage III		1.71	2.15	1.33	1.47	0.41				
Washout		1.51	1.65	2.02	1.26	0.41				
20:3 *cis*-8,*cis*-11,*cis*-14	*n-6*						0.401	0.292	0.082	0.192
Dosage III		13.04	11.26	9.41	8.59	1.61				
Washout		10.98	10.46	14.03[*]	11.13	1.63				
20:4 *cis*-5,*cis*-8,*cis*-11,*cis*-14	*n-6*						0.565	0.447	0.667	0.972
Dosage III		42.29	47.74	42.85	39.63	4.11				
Washout		40.20	40.03	44.81	38.51	4.15				
20:5 *cis*-5,*cis*-8,*cis*-11,*cis*-14,*cis*-17	*n-3*						0.624	0.518	0.135	0.011
Dosage III		6.43	7.69	7.70	6.93	0.57				
Washout		7.84	6.96	6.57	6.29	0.57				

Table S6 continued

Fatty acid (µg/g sample)	ω	CTRL[1]	CLA[2]	EFA[3]	CLA + EFA[4]	SE[5]	Treatment	Dosage	Treatment × Dosage	Time[6]
		**Treatment**					***P-value***			
22:0							0.920	0.875	0.781	0.263
Dosage III		1.13	0.97	0.86	1.07	0.42				
Washout		1.04	1.02	1.23	0.53	0.43				
22:1 *cis*-13							0.769	0.649	0.688	0.002
Dosage III		1.07	0.85	0.82	0.94	0.21				
Washout		1.05	1.08	1.05	0.77	0.22				
22:4 *cis*-7,*cis*-10,*cis*-13,*cis*-16	*n-6*						0.122	0.736	0.456	0.596
Dosage III		7.34	9.89	4.82	3.92	2.58				
Washout		5.87	6.93	7.27	4.15	2.59				
22:5 *cis*-7,*cis*-10,*cis*-13,*cis*-16,*cis*-19	*n-3*						0.503	0.227	0.108	0.575
Dosage III		6.55	9.81	12.47	12.40*	3.61				
Washout		8.41	12.61	6.76	3.85	3.63				
24:0							0.841	0.755	0.723	0.024
Dosage III		0.56	0.41	0.45	0.46	0.26				
Washout		0.32	0.53	0.64	0.15	0.26				
24:1 *cis*-15							0.453	0.420	0.224	0.000
Dosage III		0.25	0.49	0.21	0.55*	0.18				
Washout		0.23	0.50	0.36	-0.01	0.18				
SFA[9]							0.992	0.674	0.694	0.488
Dosage III		195.13	195.08	180.99	272.68	65.65				
Washout		202.22	185.20	217.22	151.24	66.38				

Table S6 continued

Fatty acid (µg/g sample)	ω	CTRL[1]	CLA[2]	EFA[3]	CLA + EFA[4]	SEM[5]	Treatment	Dosage	Treatment × Dosage	Time[6]
		**Treatment**					***P-value***			
MUFA[10]							0.981	0.600	0.918	0.180
Dosage III		244.04	250.37	243.73	265.38	25.89				
Washout		250.16	231.21	242.02	237.22	26.17				
PUFA[11]							0.679	0.779	0.886	0.889
Dosage III		301.68	316.80	302.27	283.12	35.48				
Washout		297.22	289.49	326.68	260.28	35.84				
Total CLA[12]							0.082	0.220	0.228	0.000
Dosage III		5.75	8.60	7.49	7.10[*]	1.05				
Washout		6.73	7.28	7.21	4.40	1.06				
trans-fatty acid[13]							0.969	0.453	0.731	0.084
Dosage III		9.45	8.98	10.23	14.25	3.69				
Washout		7.84	10.29	9.01	6.92	3.73				
Sum n-3 fatty acids							0.583	0.058	0.004	0.108
Dosage III		21.31[b]	27.18[ab]	37.42[*a]	35.90[*a]	4.66				
Washout		28.76	31.04	21.96	18.19	4.69				
Sum n-6 fatty acids							0.696	0.937	0.777	0.751
Dosage III		280.37	289.61	264.85	247.21	33.95				
Washout		268.46	258.45	304.72	242.09	34.30				

[1]CRTL = coconut oil (Sanct Bernhard, Bad Ditzenbach, Germany; 153.1 g/d in dosage III), mainly (93%) consist of saturated fatty acids, [2] CLA = Lutalin ® (BASF, Ludwigshafen, Germany; 64.1 g/d in dosage III; providing the same amounts of cis-9, trans-11 and trans-10, cis-12 CLA, 18.4 g/d of each in dosage III)
[3]EFA = a mix of linseed (DERBY, Derby Spezialfutter GmbH, Münster, Germany; 156.4 g/d in dosage III) and safflower oil (GEFRO, Memmingen/Allgäu, Germany; 6.4 g/d in dosage III), delivering high amounts of n-3 FA, but also some n-6 FA
[4]CLA+EFA = a combination of the EFA and CLA treatment
[5]SE = standard error of the mean; n = 4
[6]Time = statistical covariate weeks in milk
[7]Dosage III = highest dosage of specific FA supplement

[8]Washout = time period without fatty acid treatment
[9]Sum of saturated fatty acids, consisting of 8:0; 10:0; 12:0; 14:0; iso-14:0; anteiso-15:0; iso-16:0; 16:0; iso-17:0; anteiso-17:0; 18:0; 20:0; 22:0 and 24:0
[10]Sum of monounsaturated fatty acids, except of 18:1 *trans*-9 (+*trans*-6+*trans*-10) and 18:1 *trans*-11
[11]Sum of polyunsaturated fatty acids, except of 18:2, *cis*-9,*trans*-11 and 18:2, *trans*-10,*cis*-12
[12]Sum of total conjugated linoleic acids, consisting of 18:2 *cis*-9, *trans*-11and 18:2 *trans*-10, *cis*-12
[13]Sum of *trans*-fatty acids, consisting of 18:1 *trans*-9 (+*trans*-6+*trans*-10) and 18:1 *trans*-11
Within fatty acid means with different superscript letters differ among supplementation groups ($p < 0.05$)
*indicate differences between dosage III and washout period ($p < 0.05$)

Supplemental Table S7: Normalized protein abundance data of Flotillin-1 in trial 1

Cow	Week	Flotillin-1
48751	-1	2.44
29245	-1	1.67
29231	-1	1.39
52671	-1	0.67
55886	-1	1.12
48751	0	0.47
29245	0	1.32
29231	0	0.92
52671	0	0.25
55886	0	0.71
48751	1	1.54
29245	1	2.01
29231	1	1.40
52671	1	1.27
55886	1	0.21
48751	2	0.67
29245	2	0.23
29231	2	0.56
52671	2	0.59
55886	2	0.42
48751	8	0.96
29245	8	0.80
29231	8	1.24
52671	8	0.39
55886	8	0.60
48751	16	0.88
29245	16	0.55
29231	16	1.29
52671	16	0.44
55886	16	0.65
48751	24	7.69
29245	24	3.58
29231	24	3.04
52671	24	2.31
55886	24	0.89

Normalised data on flotillin-1 abundance after Western blot analysis using chemiluminescence for the detection of the specific signal. For normalization the transfer of 4 identical protein bands of each lane were quantified after Indian ink staining. The mean intensity of the 4 protein lanes were used for the normalisation of the protein data; pool samples on each blot were used to normalise different blots to each other.

Supplemental Table S8: Data on protein abundance for Flotillin-1, Pannexin-1 as well as ATP release in trial 2

Cow	Supplement	Dosage	Flotillin-1 [RU]	Pannexin-1 [RU]	ATP release [nmol/ 10^8 RBC]
607	CLA_EFA	wo	1.86	1.6	120.71
607	CLA_EFA	I			118.18
607	CLA_EFA	II			219.08
607	CLA_EFA	II	1.51	7.57	280.58
607	CLA	WO	1.53	3.82	87.83
607	CLA	I			186.31
607	CLA	II			149.16
607	CLA	III	1.84	5.2	205.65
607	EFA	WO	1.37	4.18	83.17
607	EFA	I			91.08
607	EFA	II			150.77
607	EFA	III	0.06	1.94	122.07
607	CTRL	WO	1.21	10.43	119.94
607	CTRL	I			101.24
607	CTRL	II			92.53
607	CTRL	III	2.45	7.5	164.27
128	CLA	WO	1.78	1.8	312.94
128	CLA	I			115.81
128	CLA	II			290.52
128	CLA	III	1.6	5.55	448.14
128	EFA	WO	0.6	4.27	180.73
128	EFA	I			359.15
128	EFA	II			282.88
128	EFA	III	0.91	7.25	473.46
128	CTRL	WO	1.46	7.76	262.13
128	CTRL	I			157.7
128	CTRL	II			214.01
128	CTRL	III	0.19	5.16	247.96
128	CLA_EFA	WO	0.86	8.57	183.47
128	CLA_EFA	I			173.22
128	CLA_EFA	II			193.35
128	CLA_EFA	III	1.46	7.97	267.93
524	CTRL	WO	1.6	2.44	139.76
524	CTRL	I			221.38
524	CTRL	II			269.54
524	CTRL	III	2.06	3.7	368.81

Table S8 continued

Cow	Supplement	Dosage	Flotillin-1 [RU]	Pannexin-1 [RU]	ATP release [nmol/ 10^8 RBC]
524	CLA_EFA	WO	0.91	4.53	166.46
524	CLA_EFA	I			365.45
524	CLA_EFA	II			200.24
524	CLA_EFA	III	1.33	4.14	307.68
524	CLA	WO	3.21	3.81	105.66
524	CLA	I			112.33
524	CLA	II			178.72
524	CLA	III	0.8	3.8	174.73
524	EFA	WO	1.5	5.66	219.24
524	EFA	I			137.82
524	EFA	II			143.33
524	EFA	III	4.22	5.14	227.71
624	EFA	WO	0.57	2.27	142.05
624	EFA	I			202.55
624	EFA	II			240.25
624	EFA	III	0.59	3.25	365.26
624	CTRL	WO	0.6	3.52	119.4
624	CTRL	I			208.34
624	CTRL	II			205.19
624	CTRL	III	2.03	4.98	277.03
624	CLA_EFA	WO	0.32	9.35	125.92
624	CLA_EFA	I			107.95
624	CLA_EFA	II			158.36
624	CLA_EFA	III	0.58	4.67	145.78

Normalised data on flotillin-1 and pannexin-1 abundance after Western blot analysis using chemiluminescence for the detection of the specific signal. For normalization the transfer of 4 identical protein bands of each lane were quantified after Indian ink staining. The mean intensity of the 4 protein lanes were used for the normalisation of the protein data; pool samples on each blot were used to normalise different blots to each other. ATP release was analysed in vitro using luminescence for the detection of the signal and was normalised to 10^8 red blood cells (RBC) after counting the cell numbers of the three dilution series, which were analysed for each sample.

Supplemental Table S9: Data on haematocrit values of the 2% dilution used for the measurement of ATP in trial 2

	Cow				
**Sampling date**	**Blank**	**27624**	**27524**	**29128**	**38607**
04.06.2015	0.047	0.067	0.067	0.057	0.047
	0.049	0.068	0.039	0.062	0.047
	0.039	0.056	0.051	0.057	0.053
25.06.2015	0.055	0.059	0.047	0.052	0.052
	0.039	0.061	0.053	0.053	0.054
	0.054	0.063	0.066	0.065	0.054
08.07.2015	0.058	0.061	0.06	0.061	0.054
	0.055	0.061	0.06	0.061	0.068
	0.045	0.052	0.05	0.054	0.049
22.07.2015	0.046	0.051	0.043	0.053	0.046
	0.013	0.018	0.02	0.024	0.005
05.08.2015	0.014	0.018	0.017	0.037	0.005
	0.055	0.058	0.057	0.062	0.061
09.09.2015	0.05	0.058	0.052	0.059	0.062
	0.05	0.057	0.057	0.062	0.059
	0.049	0.056	0.059	0.062	0.058
23.09.2015	0.046	0.057	0.059	0.06	0.056
	0.047	0.055	0.059	0.061	0.059
	0.049	0.056	0.053	0.054	0.057
07.10.2015	0.046	0.053	0.045	0.051	0.052
	0.047	0.055	0.053	0.051	0.054
	0.042	0.085	0.061	0.06	0.055
28.10.2015	0.042	0.088	0.056	0.058	0.053
	0.044	0.081	0.062	0.058	0.056
	0.046	0.044	0.044	0.049	0.071
11.11.2015	0.043	0.044	0.039	0.045	0.18
	0.044	0.044	0.044	0.044	0.195
	0.044	0.053	0.046	0.054	0.048
25.11.2015	0.045	0.051	0.048	0.047	0.044
	0.045	0.051	0.047	0.047	0.046
	0.054	0.064	0.058	0.061	0.053
09.12.2015	0.047	0.064	0.057	0.064	0.054
	0.046	0.063	0.056	0.064	0.053

In a first step to analyse the ATP release from red blood cells (RBC) cells were stepwise diluted to a haematocrit of 2%. For measurement of ATP, the 2% RBC were further diluted into three concentrations (0.04%, 0.02% and 0.01%). To ensure the quality of RBC used for the measurements, free haemoglobin was analysed in the 2% RBC dilutions in comparison to the washing buffer at 415 nm (SunriseTM, TECAN, Switzerland). The maximum absorbance value of the bovine haemoglobin was determined before the beginning of the experiment.

Supplemental Figure S1: Exemplarily representation of Western blots for flotillin-1 (trial 1)

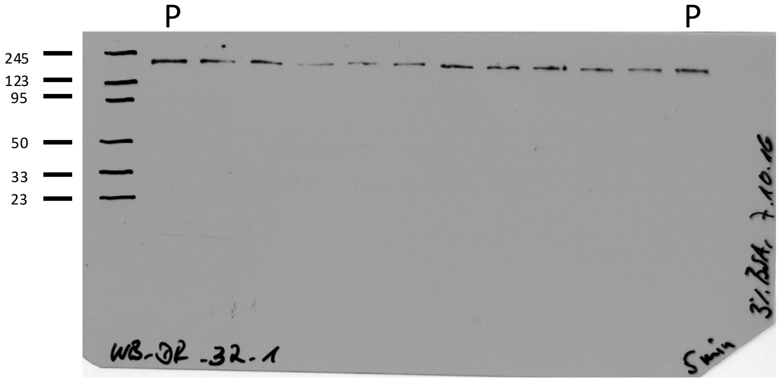

Cow	Week	Lane
48751	2	3-4
29245	2	5-6
29231	2	7-8
52671	2	9-10
55886	2	11-12

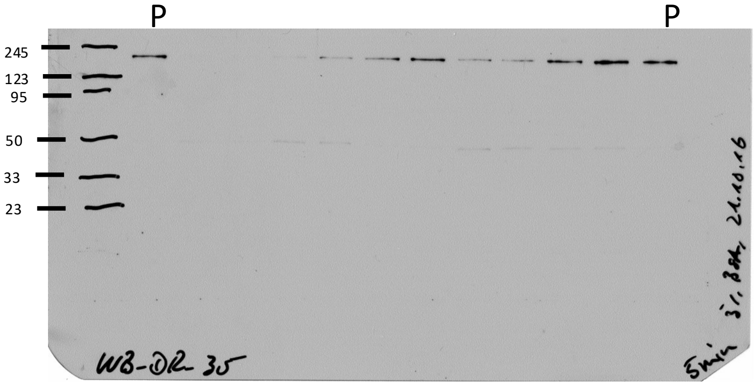

Cow	Week	Lane
52761	-1	3-4
55886	1	5-6
29231	8	7-8
52671	8	9-10
55886	24	11-12

Demonstration of a Western blot for pannexin-1 (trial 1) followed by the corresponding sample loading table. P represents a sample pool, which was used for the normalisation between different blots. Mostly multimers were detected and quantified by the used protocol.

Supplemental Figure S2:

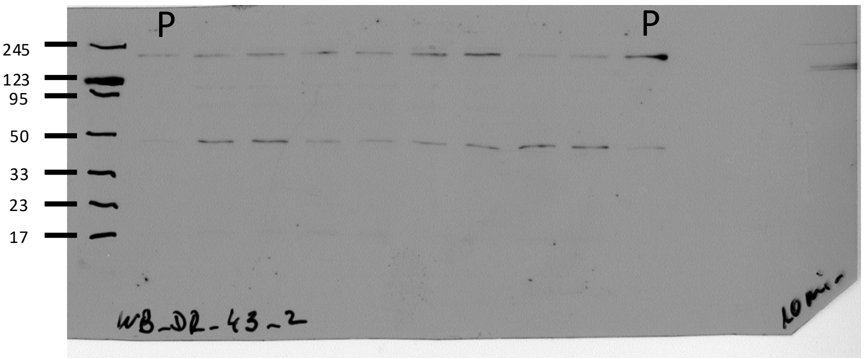

Cow	Fatty acid	Lane
27625	CTRL	3-4
27254	EFA_CLA	5-6
29128	EFA	7-8
38607	CLA	9-10

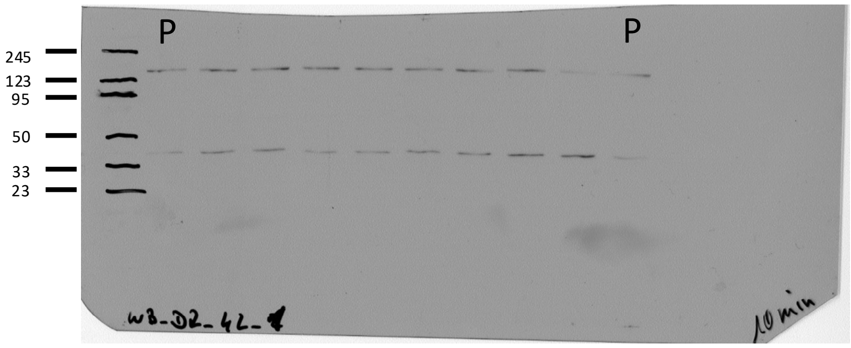

Cow	Fatty acid	Lane
27625	WO	3-4
27254	WO	5-6
29128	WO	7-8
38607	WO	9-10

Demonstration of two Western blot for flotillin-1 (trial 2) followed by the corresponding sample loading table after each blot. Multimers and monmers of flotillin-1 were observed by the used protocol. Abbreviations: CRTL = coconut oil (Sanct Bernhard, Bad Ditzenbach, Germany; 153.1 g/d in dosage III), mainly (93%) consist of saturated fatty acids, CLA = Lutalin ® (BASF, Ludwigshafen, Germany; 64.1 g/d in dosage III; providing the same amounts of cis-9, trans-11 and trans-10, cis-12 CLA, 18.4 g/d of each in dosage III); EFA = a mix of linseed (DERBY, Derby Spezialfutter GmbH, Münster, Germany; 156.4 g/d in dosage III) and safflower oil (GEFRO, Memmingen/Allgäu, Germany; 6.4 g/d in dosage III), delivering high amounts of n-3 FA, but also some n-6 FA; P represents a sample pool, which was used for the normalisation between different blots.

Supplemental Figure S3:

A

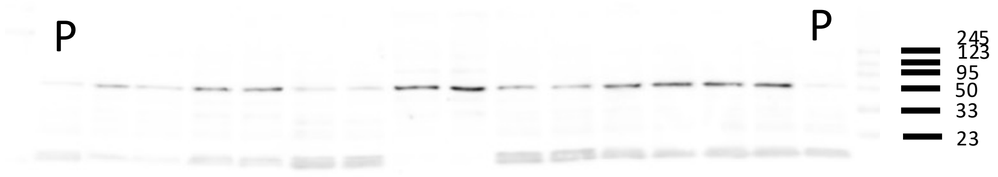

B

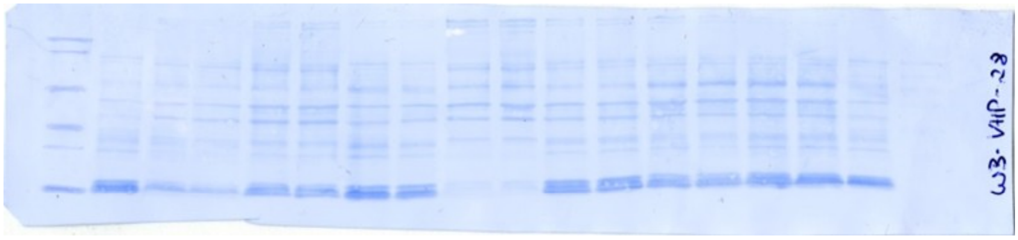

C

Cow	Fatty acid	Lane
38607	CLA	3-4
29128	WO	5-6
38607	EFA	7-8
27624	WO	9-10
27524	WO	11-12
38607	WO	13-14
38607	CTRL	15-16

Demonstration of a Western blot for pannexin-1 (trial 2). A: Western blot; B: Corresponding Indian ink staining, C: Corresponding sample loading with cow number and the fatty acid supplement. Abbreviations:
CRTL = coconut oil (Sanct Bernhard, Bad Ditzenbach, Germany; 153.1 g/d in dosage III), mainly (93%) consist of saturated fatty acids; CLA = Lutalin ® (BASF, Ludwigshafen, Germany, 64.1 g/d in dosage III, providing the same amounts of cis-9, trans-11 and trans-10, cis-12 CLA, 18.4 g/d of each in dosage III); EFA = a mix of linseed (DERBY, Derby Spezialfutter GmbH, Münster, Germany; 156.4 g/d in dosage III) and safflower oil (GEFRO, Memmingen/Allgäu, Germany; 6.4 g/d in dosage III), delivering high amounts of n-3 FA, but also some n-6 FA; P represents a sample pool, which was used for the normalisation between different blots.
